# Supplementary material for: Streptococcus pneumoniae serotype 33G: genetic, serological, and structural analysis of a new capsule type
Source: Microbiol Spectr. 2023 Dec 7;12(1):e03579-23. doi: 10.1128/spectrum.03579-23 (PMC10782959; doi:10.1128/spectrum.03579-23)
Supplement: Supplemental material — Fig. S1 to S7; Tables S1 and S2. [file spectrum.03579-23-s0001.docx]

**Supplementary data:**

***Streptococcus pneumoniae* serotype 33G:**

**genetic, serological, and structural analysis of a new capsule type**

Sam Manna, Joel P. Werren, Belinda D. Ortika, Barbara Bellich, Casey L. Pell, Elissavet Nikolaou, Ilche Gjuroski, Stephanie Lo, Jason Hinds, Odgerel Tundev, Eileen M. Dunne, Bradford D. Gessner, Stephen D. Bentley, Fiona M. Russell, E. Kim Mulholland, Tuya Mungun, Claire von Mollendorf, Paul V. Licciardi, Paola Cescutti, Neil Ravenscroft, Markus Hilty & Catherine Satzke

Y5914-11_acyltransferase ATGAAGAAGGAATATGATATTTTAAAAGTAATTGCCATTTTAATGGTTGTATTAAGCCAC 60

33X_orf3/4region ATGAAGAAGGAATATGATATTTTAAAAGTAATTGCCATTTTAATGGTTGTATTAAGCCAC 60

************************************************************

Y5914-11_acyltransferase AGTACATACTATGTGATTTCGACTAAGTACGGGGGGGTTGATTATCAACAATATATAAAT 120

33X_orf3/4region AGTACATACTATGTGATTTCGACTAAGTACGGGGGGA----TTATCAACAATATATAAAT 116

************************************. *******************

Y5914-11_acyltransferase CAAAATTTATCGTTGGTATTGTATAAAGTTTTTGATAAAGTAAGAGAAGTATTATATTAC 180

33X_orf3/4region CAAAATTTATCGTTGGTATTGTATAAAGTTTTTGATAAAGTAAGAGAAGTATTATATTAC 176

************************************************************

Y5914-11_acyltransferase TTCCATATGCCACTTTTTATGGCATTGTCAGGAGCTTTCTACTATCTTCAGGTTCAAAGA 240

33X_orf3/4region TTCCATATGCCACTTTTTATGGCATTATCAGGAGCTTTCTACTATCTTCAGGTTCAAAGA 236

**************************.*********************************

Y5914-11_acyltransferase GATAAATGGTCTACTTTGAAATTAATTGTGCAAAA-----TAAGGCAAAGAGATTGCTTT 295

33X_orf3/4region GATAAATGGTTTACTTTAAAATTAATTGTGCAAAATAAAATAAGGTAAAGAGATTGCTTT 296

********** ******.***************** ***** **************

Y5914-11_acyltransferase TTCCTTTCATTATATTTACTATTCTTTATTCAATACCAATAAAATATATTTCAAATTATT 355

33X_orf3/4region TTCCTTTCATTATATTTACTGTCCTTTATTCAATACCAATAAAATATATTTCAAATTATT 356

********************.* *************************************

Y5914-11_acyltransferase TTGATTCTACAGATCCTTTTAAAGCATTTGTAGGACAATTTTTCTTAATTGGGAATAGTC 415

33X_orf3/4region TTGATTTTACAGCTCCTTTTAAAGCATTTGTAGGAGAATTTTTCTTAATTGGAAATAGTC 416

****** *****.********************** ****************.*******

Y5914-11_acyltransferase ATTTATGGTATTTATATGCTTTATTTATTATTTTTATATTTGCATTCTATACGCTAAAAA 475

33X_orf3/4region ATTTATGGTATTTATATGCTTTATTTATTATTTTTATATTTGCATTCTATACGCTAAA-A 475

********************************************************** *

Y5914-11_acyltransferase AAGAAACAAAGCTTGCTACTTATGTTGTCTTTTATGTTCTGCATATTTTGAGTTACAAAA 535

33X_orf3/4region AAGAAACAAATCTTGCTACTTATGTTGTTTTTTATGTTCTGTATATTTTGAGTTACAAAA 535

********** ***************** ************ ******************

Y5914-11_acyltransferase TAGAACTCCCACTATTTAAAGTACCTCTTCAATTTTTATTTTACTTTAGTTTAGGCTTTT 595

33X_orf3/4region TAGAACTCACACTATTTAAAGTACCTCTTCAATTTTTATTTTACTTTAGTTTAGGCTTCT 595

********.************************************************* *

Y5914-11_acyltransferase TGTTTGAATCAAATAGAGAAAAATATAATCAATTTATTAATAAGAAAAAAAATTATGTTT 655

33X_orf3/4region TGTTTGAATCAAATAGAGAAAAATATAATCAATTTATTAATAGGAAA-AAAATTATATTT 654

******************************************.**** ********.***

Y5914-11_acyltransferase TGTTATTATCTACGGTATTTGCCTTAATGGTTTTATTAAATCTCTATGTTAAGTTTACTC 715

33X_orf3/4region TGTTATTATCTACGGTATTTGTCTTAATGGTTTTG-------------TTAAATTTACTC 701

********************* ************. ****.*******

Y5914-11_acyltransferase AGGTATTGTTTAGTAAAGTATTAGTTGAGTTAATGGCTGTCTTGGGTTCATTATTAACGT 775

33X_orf3/4region AGGTATTTTTTAGTAAAGTATTAGTTGAGCTATTGGCTGTCTTGGGTTCATTATTAACGT 761

******* ********************* **:***************************

Y5914-11_acyltransferase ATAGCATCGCGTATCAATTATCTCAAAAGAAAAGTGTTGGTGATGCTAGTATTTTTAAGA 835

33X_orf3/4region ATAGCATCGCATATCAATTATCTCAAAAGAAAAGTGTTGGTGATGCTAGTATTTTTAAGA 821

**********.*************************************************

Y5914-11_acyltransferase TGATTCTAATTAATGGATTAGGTATATATATTTTTTCTGATCCATTAAATTATTTAATCT 895

33X_orf3/4region TGATTCTAATTAATGGATTAGGTATATATATTTTTTCTGATCCATTAAATTATTTAATCT 881

************************************************************

Y5914-11_acyltransferase TGAAATTAAGTTATTCCCTAAATTCGTATTTTATGTTTACACCATTAGGAATAGTTGTTT 955

33X_orf3/4region TGAAGTTAAGTTATTCCCTAAATTCGTATTTTATGTTTACACCAGTAGGAATAATTGTTT 941

****.*************************************** ********.******

Y5914-11_acyltransferase TAGTGGTGCTACGATTTTTTCTTACGTTATTTATATCATTGATAGGAACAATTATATTTA 1015

33X_orf3/4region TAGTAGTATTACGATTTTTTCTCACGTTATTCATATCATTGATAGGAACAATCATATTT- 1000

****.**. ************* ******** ******************** ******

Y5914-11_acyltransferase AAAAATATTTTAAAAAATACAAATGGCTAGTCAACTAG---------------------- 1053

33X_orf3/4region ----------AAAAAAATACAAATGGCTAGTCAACTAGGTAGAGACAATAGCTATTTAAA 1050

:***************************

Y5914-11_acyltransferase ------- 1053

33X_orf3/4region AAGTTAA 1057

Figure S1: DNA sequence alignment of the acyltransferase gene from *S. oralis* subsp. *Dentisani* strain Y5914-11 (Genbank accession NZ_NCUW01000028, locus tag B7709_03985) with the *orf3-orf4* region from the pneumococcal 33X *cps* locus. Red and blue text denote the predicted *orf3* and *orf4* sequences detected in the 33X *cps* locus. Nucleotides highlighted in yellow denote stop codons created as a result of frameshift mutations.


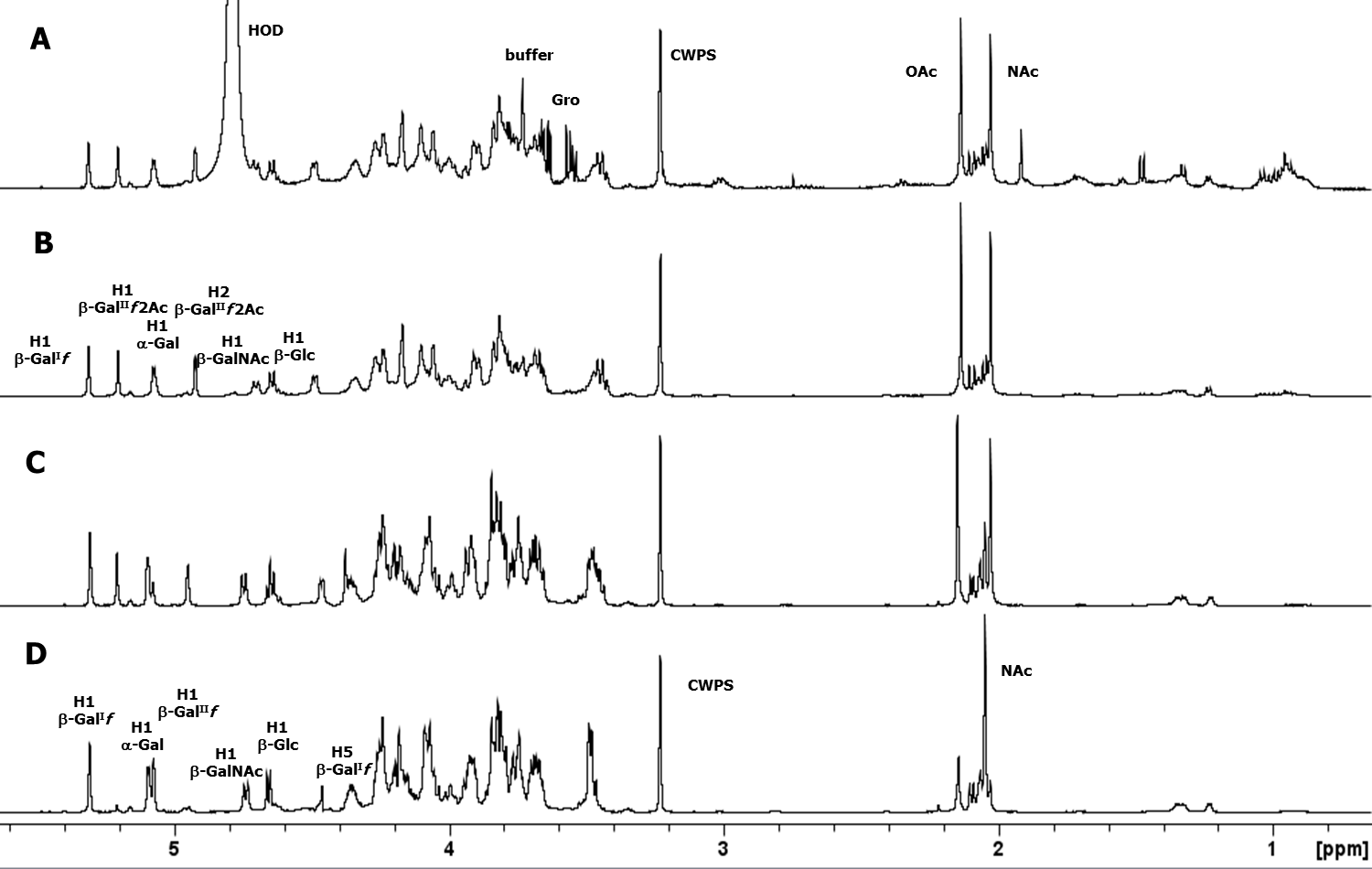


Figure S2: 1D proton NMR spectra of pneumococcal serotype 33G polysaccharide, some diagnostic anomeric and ring protons are labeled. (A) 1D 1H NMR at 500 MHz and 25°C, (B) 1D DOSY at 500 MHz and 25°C, (C) 1D DOSY at 600 MHz and 70°C (partially de-O-acetylated), and (D) 1D DOSY at 600 MHz and 60°C (de-O-acetylated polysaccharide). The 1D DOSY (Figure 3D) gave five anomeric signals of similar intensity at *δ* 5.31 (br s), 5.09 (d, ^3^*J*_H-1,H-2_ = 3.6 Hz), 5.07 (br s), 4.74 (d, ^3^*J*_H-1,H-2_ = 8.4 Hz) and 4.66 (d, ^3^*J*_H-1,H-2_ = 7.8 Hz) consistent with a hexasaccharide repeat unit. The broad singlets were attributed to β-galactofuranose residues (confirmed by the presence of characteristic deshielded C1 resonances at 108.5 and 109.3 ppm, Figure S4), whereas the chemical shift and vicinal coupling constant values of the remaining signals were in a good agreement with an α-pyranose and two β-pyranose sugars.


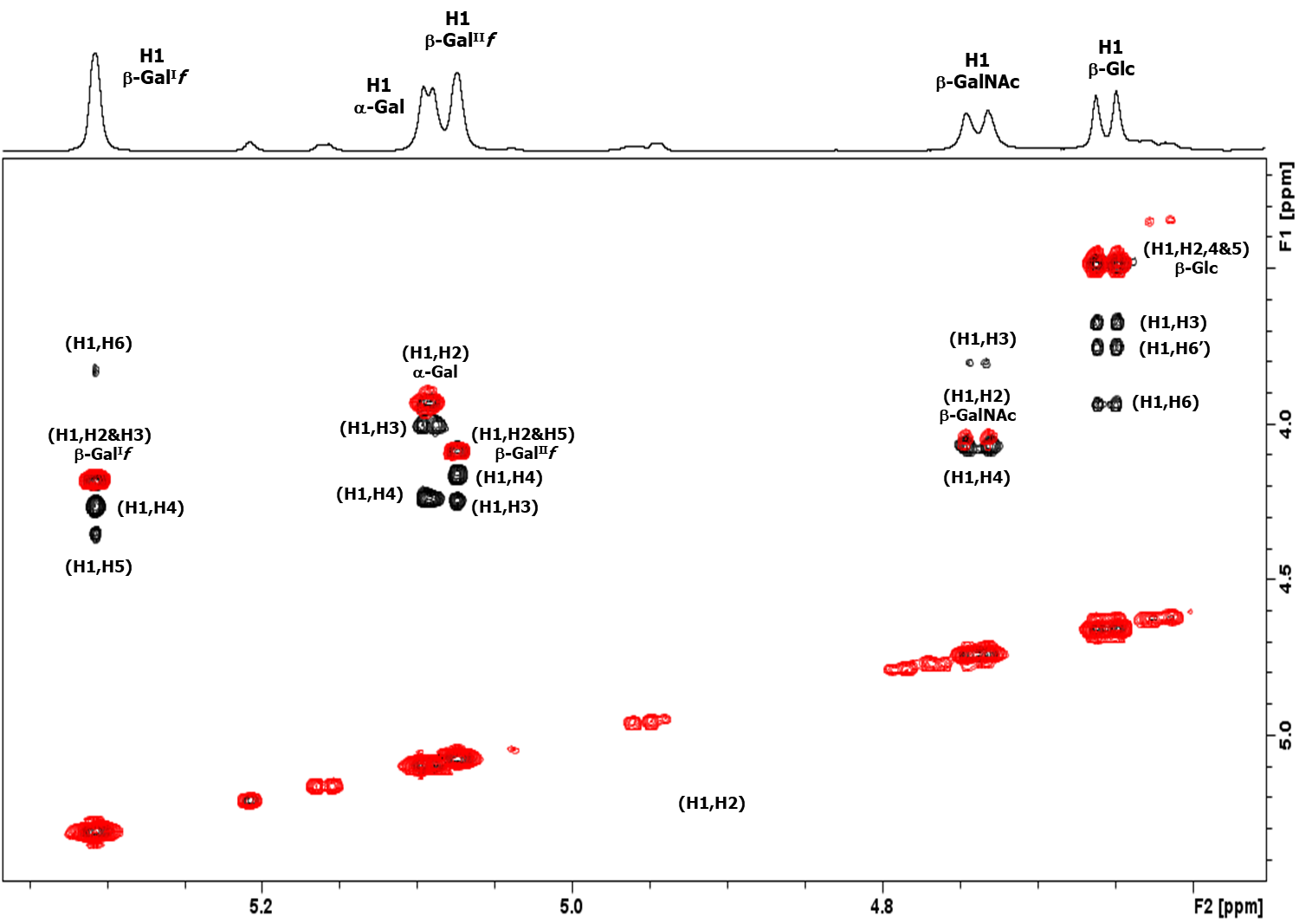


Figure S3: The 2D ^1^H-^1^H overlay for polysaccharide 33G: COSY (red)/ TOCSY (black) recorded at 600 MHz (60°C). The major crosspeaks from H1 for the 33G repeat unit are labeled. The DOSY-TOCSY experiment was recorded using a mixing time of 180 ms. The COSY spectrum gave H1 to H2 for each residue and the TOCSY experiment established further correlations for each spin system depending on the sugar type: H1 to H6 for β-Gal^I^*f*, H1 to H4 for α-Gal*p*, H1 to H5 for β-Gal^II^*f*, H1 to H4 for β-GalNAc and H1 to H6 for β-Glc. The assignments were confirmed, and some additional protons identified using 1D TOCSY profiles (Figure S3).


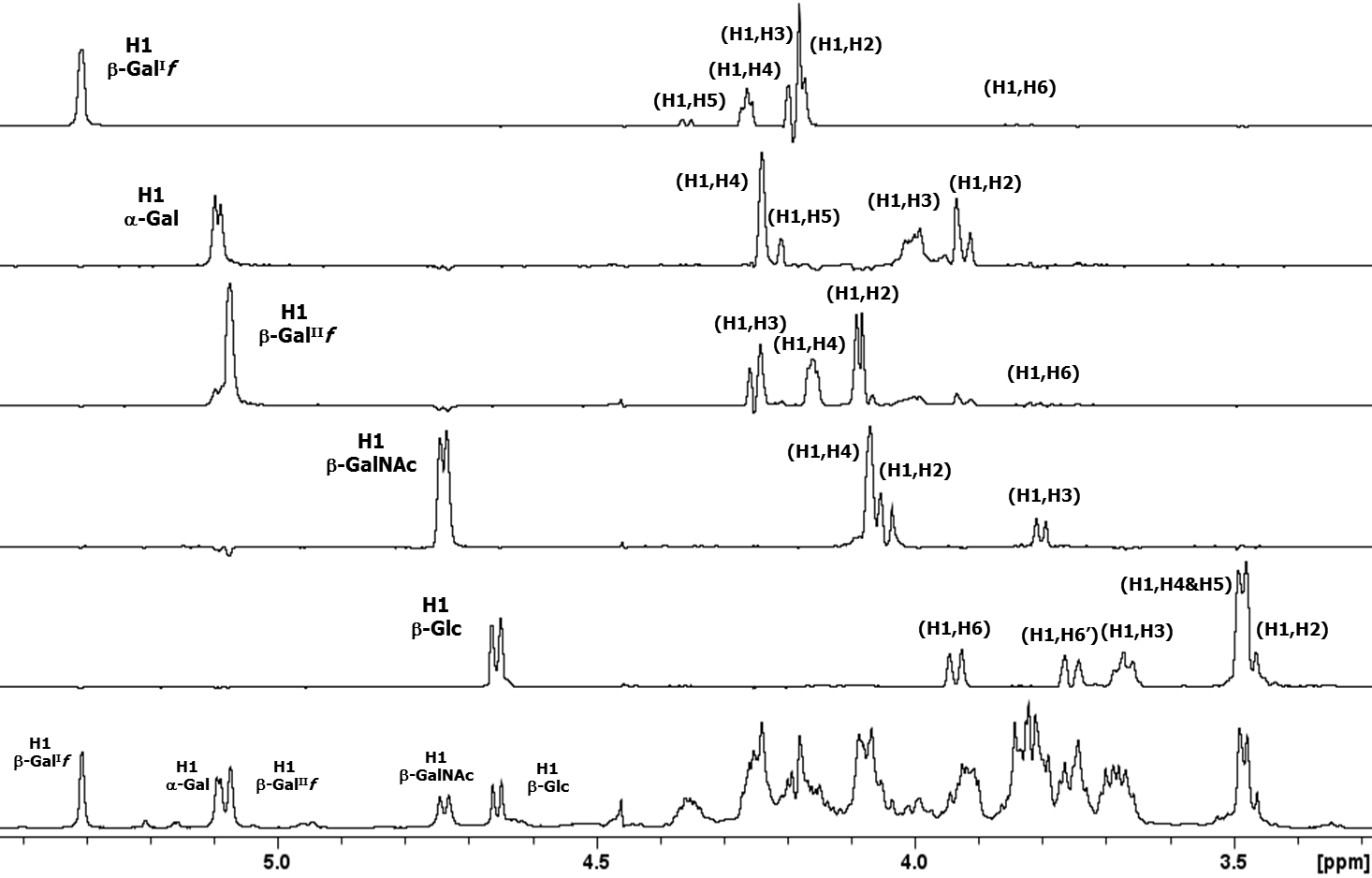


Figure S4: Overlay of 1D TOCSY (180 ms) profiles extracted from the 2D DOSY-TOCSY experiment overlaid over the 1D DOSY spectrum. The spectra corroborated the 2D NMR assignments (Figure S2).


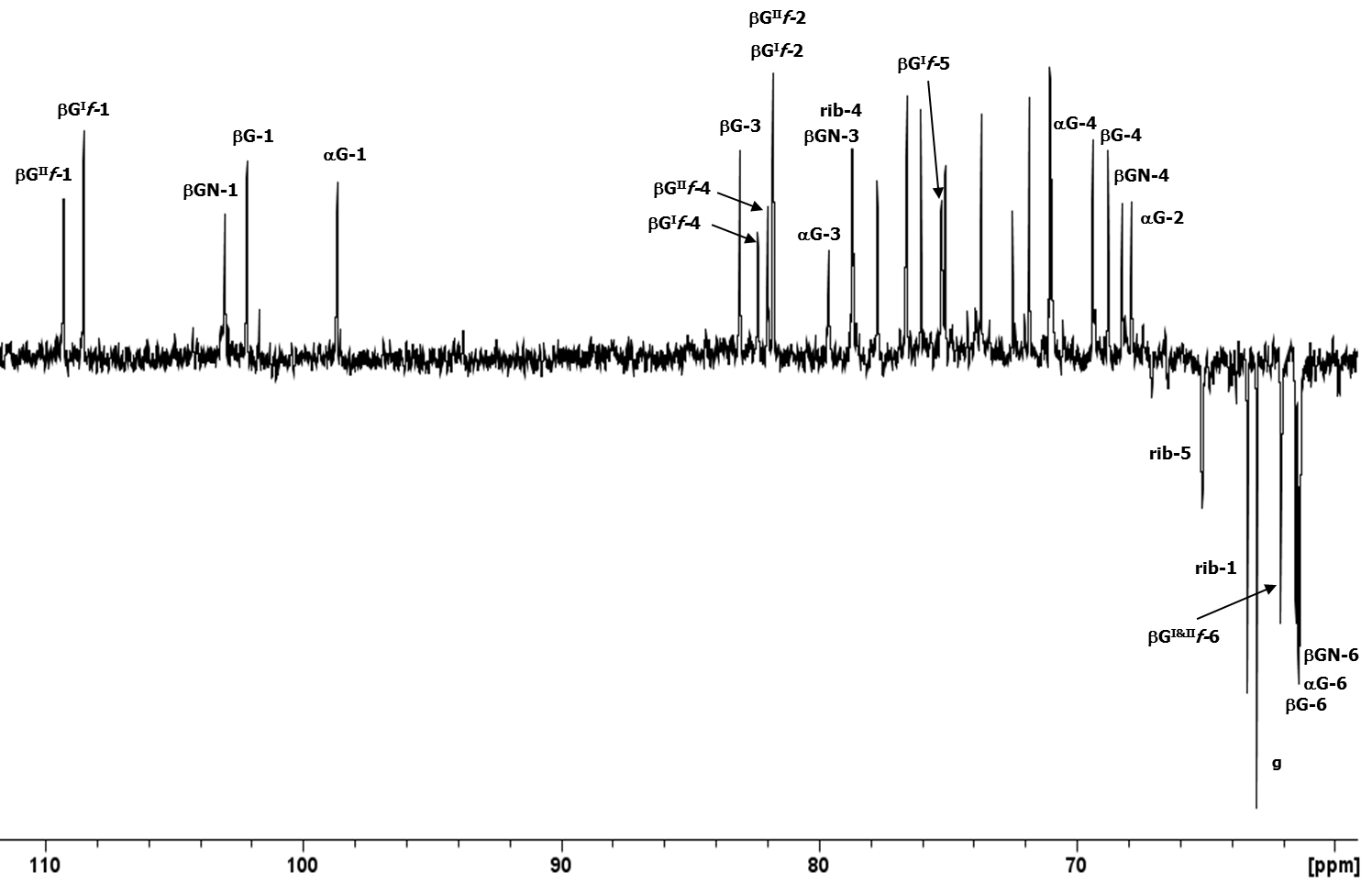


Figure S5: Expansion of the ^13^C DEPT-135 spectrum of polysaccharide 33G recorded at 150 MHz and 60°C. Diagnostic peaks from the hexasaccharide repeating unit have been labeled (βG^I^*f* = β-Gal^I^*f*, αG = α-Gal*p*, βG^II^*f* = β-Gal^II^*f*, βGN = β-GalNAc, β-G = β-Glc and rib = ribitol). Additional peaks are due to glycerol (g). The presence of ribitol-5- phosphate was confirmed by the methylene peaks at *δ* 63.4 (H1s at 3.83 and 3.68 ppm) and 65.1 ppm (H5s at 4.21 and 4.14 ppm) and splitting of the latter by coupling with ^31^P (*J*_H,P_ = 5.5 Hz).


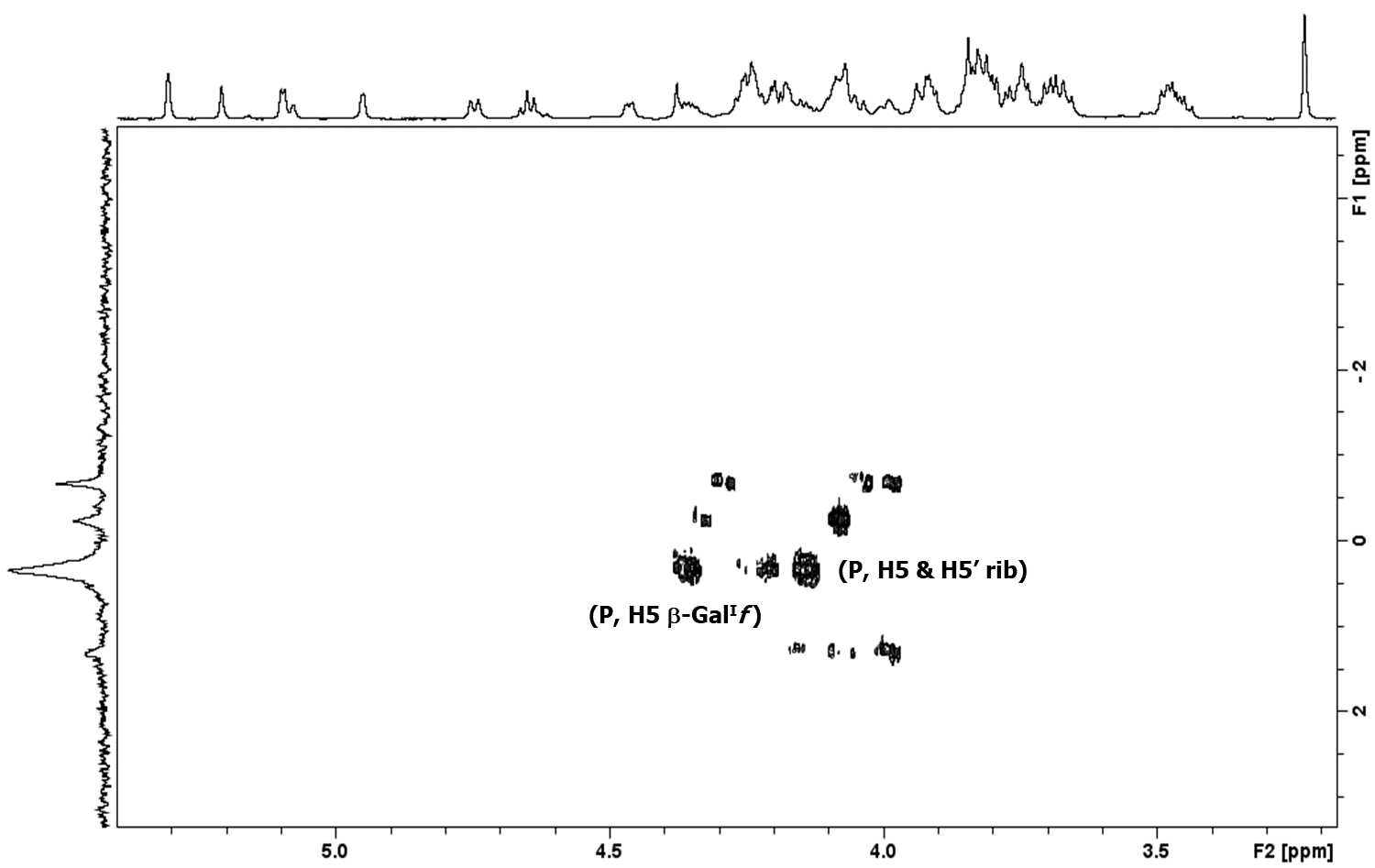


Figure S6. The 2D ^1^H–^31^P HMBC spectrum for polysaccharide 33G recorded at 600 MHz (70°C). The major crosspeaks from the phosphodiester at 0.34 ppm are labeled (H5s of ribitol and H5 of β-Gal^I^*f*); the additional unlabeled peaks are from residual CWPS. The HMBC experiment was optimized for a coupling constant of 10 Hz.


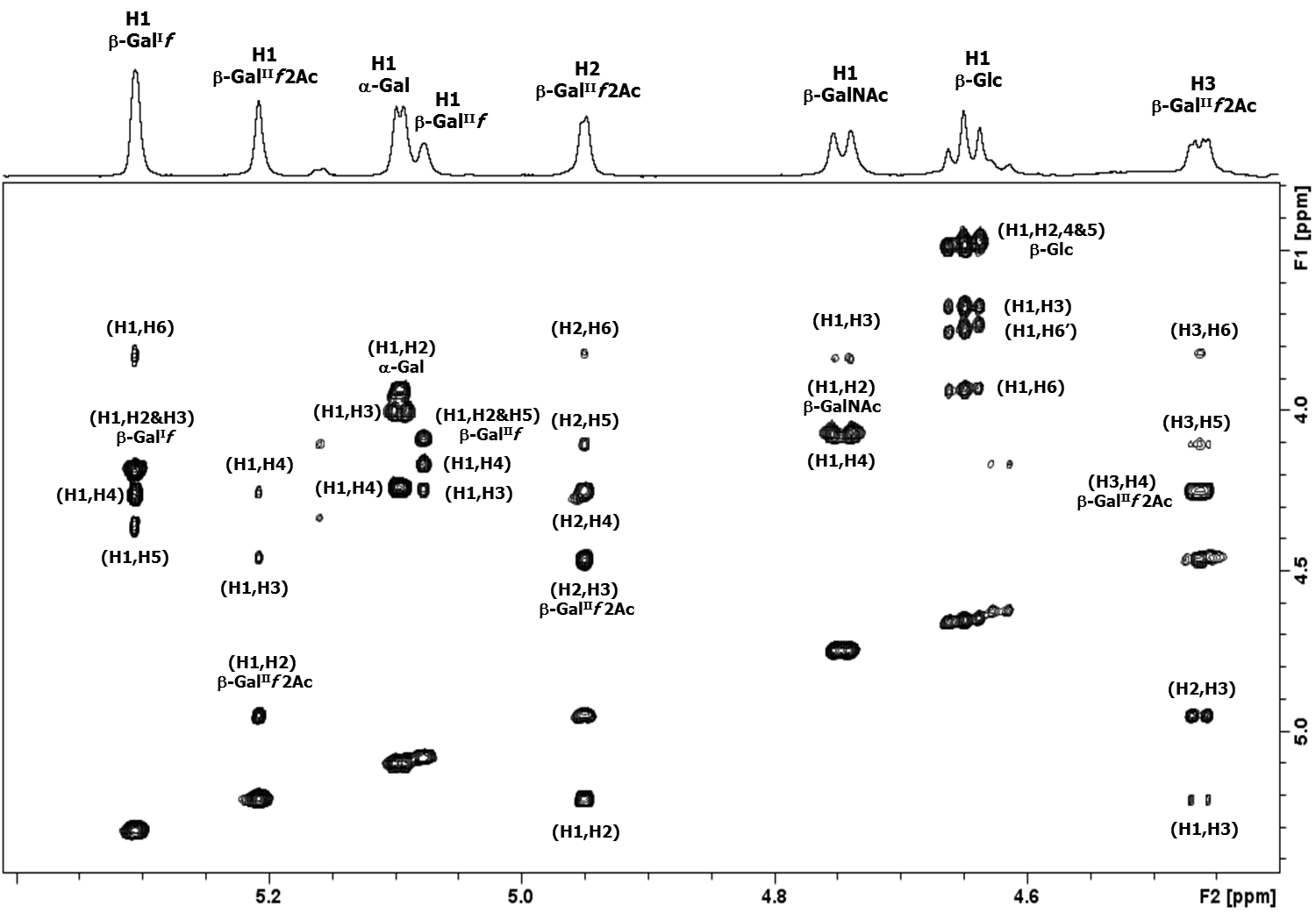


Figure S7: The 2D ^1^H-^1^H TOCSY of the partially O-acetylated 33G polysaccharide recorded at 600 MHz (70°C). The major crosspeaks from H-1 and other diagnostic peaks in the anomeric region for the 33G repeat unit are labeled.

Table S1. Pneumococcal-positive nasopharyngeal swabs containing 33X pneumococci in Mongolia

| **Year** | **Pneumococcal-positive swabs containing serotype 33X**  **(n/N, %)** |
| --- | --- |
| 2015 | 0/701 (0%) |
| 2016 | 0/600 (0%) |
| 2017 | 0/1006 (0%) |
| 2018 | 3/688 (0.4%) |
| 2019 | 6/982 (0.6%) |
| 2020 | 7/462 (1.5%) |
| 2021^a^ | 0/33 (0%) |
| 2022 | 4/328 (1.2%) |

^a^ Overall sample numbers in 2021 were very low consistent with the non-pharmaceutical interventions in place for the COVID-19 pandemic

Table S2. Summary of molecular serotyping results for 33X pneumococci

|  | **DNA microarray** (1) | **PneumoCaT** (2) | **seroBA** (3) | **serocall** (4) | **PneumoKITy** (5) | **PfaSTer** (6) | **TaqMan Array Card** (7) |
| --- | --- | --- | --- | --- | --- | --- | --- |
| **Serotype result** | 35A/10B-like^a^ | Non-typeable | 10X | Non-typeable | Non-typeable | Non-typeable (Mean probability = 0.17) | 10B |
| **Best serotype match** | N/A | 35B  (Mean coverage = 59%) | N/A | N/A | 35B  (Mean hash score = 44%) | 35D  (low confidence) | N/A |

^a^a ‘like’ designation indicates that DNA microarray could not designate to a known serotype, but has sequence similarity to portions of the serotype 35A and 10B *cps* loci

**References**

1. Satzke C, Dunne EM, Porter BD, Klugman KP, Mulholland EK, Vidal JE, Sakai F, Strachan JE, Hay Burgess DC, Holtzman D, Boelsen K, Habib M, Manning J, Ortika BD, Pell CL, Smyth JA, Antonio M, Klugman KP, O’Brien KL, Robins-Browne RM, Anthony Scott J, Saha SK, Russell FM, Greenhill AR, Lehmann D, Adrian P V., Madhi SA, Rubin LG, Rizvi A, Hinds J, Gould KA, Kong F, Oftadeh S, Gilbert GL, Feng L, Cao B, Paranhos-Baccalà G, Telles JN, Messaoudi M, Borrow R, Stanford E, George R, Slotved HC, Brugger SD, Mühlemann K, Hilty M, Rivera-Olivero IA, de Waard JH, Charalambous BM, Leung MH, Azzari C, Moriondo M, Nieddu F, Hermans PWM, van der Gaast-de Jongh CE, Turner P, Ecker DJ, Sampath R. 2015. The PneuCarriage project: A multi-centre comparative study to identify the best serotyping methods for examining pneumococcal carriage in vaccine evaluation studies. PLoS Med 12:e1001903.

2. Kapatai G, Sheppard CL, Al-Shahib A, Litt DJ, Underwood AP, Harrison TG, Fry NK. 2016. Whole genome sequencing of *Streptococcus pneumoniae*: development, evaluation and verification of targets for serogroup and serotype prediction using an automated pipeline. PeerJ 4:e2477.

3. Epping L, van Tonder AJ, Gladstone RA, Bentley SD, Page AJ, Keane JA. 2018. SeroBA: Rapid high-throughput serotyping of *Streptococcus pneumoniae* from whole genome sequence data. Microb Genomics 4:e000186.

4. Knight JR, Dunne EM, Mulholland EK, Saha S, Satzke C, Tothpal A, Weinberger DM. 2021. Determining the serotype composition of mixed samples of pneumococcus using whole-genome sequencing. Microb Genomics 7:1–10.

5. Sheppard CL, Manna S, Groves N, Litt DJ, Amin-Chowdhury Z, Bertran M, Ladhani S, Satzke C, Fry NK. 2022. PneumoKITy: A fast, flexible, specific, and sensitive tool for Streptococcus pneumoniae serotype screening and mixed serotype detection from genome sequence data. Microb Genomics 8:000904.

6. Lee JT, Li X, Hyde C, Liberator PA, Hao L. 2023. PfaSTer: a machine learning-powered serotype caller for *Streptococcus pneumoniae* genomes. Microb Genomics 9:001033.

7. Pholwat S, Sakai F, Turner P, Vidal JE, Houpt ER. 2016. Development of a taqman array card for pneumococcal serotyping on isolates and nasopharyngeal samples. J Clin Microbiol 54:1842–1850.
